# The Raphe Pallidus and the Hypothalamic-Pituitary-Thyroid Axis Gate Seasonal Changes in Thermoregulation in the Hibernating Arctic Ground Squirrel (*Urocitellus parryii*)

**DOI:** 10.3389/fphys.2018.01747

**Published:** 2018-12-12

**Authors:** Carla Frare, Mackenzie E. Jenkins, Steven J. Soldin, Kelly L. Drew

**Affiliations:** ^1^Department of Chemistry and Biochemistry, University of Alaska Fairbanks, Fairbanks, AK, United States; ^2^Institute of Arctic Biology, University of Alaska Fairbanks, Fairbanks, AK, United States; ^3^National Institutes of Health Clinical Center, Bethesda, MD, United States; ^4^Division of Endocrinology and Metabolism, Department of Medicine, Georgetown University, Washington, DC, United States

**Keywords:** season, hibernation, thyroid hormones, thermoregulation, thermogenic capacity, SNS, raphe pallidus, adenosine

## Abstract

Thermoregulation is necessary to maintain energy homeostasis. The novel discovery of brown adipose tissue (BAT) in humans has increased research interests in better understanding BAT thermogenesis to restore energy balance in metabolic disorders. The hibernating Arctic ground squirrel (AGS) offers a novel approach to investigate BAT thermogenesis. AGS seasonally increase their BAT mass to increase the ability to generate heat during interbout arousals. The mechanisms promoting the seasonal changes in BAT thermogenesis are not well understood. BAT thermogenesis is regulated by the raphe pallidus (rPA) and by thyroid hormones produced by the hypothalamic–pituitary–thyroid (HPT) axis. Here, we investigate if the HPT axis and the rPA undergo seasonal changes to modulate BAT thermogenesis in hibernation. We used histological analysis and tandem mass spectrometry to assess activation of the HPT axis and immunohistochemistry to measure neuronal activation. We found an increase in HPT axis activation in fall and in response to pharmacologically induced torpor when adenosine A_1_ receptor agonist was administered in winter. By contrast, the rPA neuronal activation was lower in winter in response to pharmacologically induced torpor. Activation of the rPA was also lower in winter compared to the other seasons. Our results suggest that thermogenic capacity develops during fall as the HPT axis is activated to reach maximum capacity in winter seen by increased free thyroid hormones in response to cooling. However, thermogenesis is inhibited during torpor as sympathetic premotor neuronal activation is lower in winter, until arousal when inhibition of thermogenesis is relieved. These findings describe seasonal modulation of thermoregulation that conserves energy through attenuated sympathetic drive, but retains heat generating capacity through activation of the HPT axis.

## Introduction

Thermoregulation is necessary to maintain energy homeostasis and when disrupted can lead to metabolic disorders. The novel discovery of brown adipose tissue (BAT) in humans (Betz and Enerbäck, 2015) has increased research interests in better understanding BAT thermogenesis to develop therapeutic approaches to increase energy expenditure activating BAT to treat obesity and diabetes (Betz and Enerbäck, 2015). The phenomenon of hibernation is an innovative model to investigate overall energy homeostasis and BAT thermogenesis, since this evolutionary adaptation is a model of physiological obesity (Cannon and Nedergaard, 2004) without any health consequences seen in humans such as type 2 diabetes and heart disease.

Hibernation is a seasonal phenomenon that is preceded by a fall transition phase where body weight increases by 40–60% (Sheriff et al., 2013; MacCannell et al., 2017) and body temperature (*T*_b_) decreases 1–2°C from summer euthermic *T*_b_ (Russell et al., 2010; Sheriff et al., 2012). During hibernation, metabolic rate is suppressed to 1–2% of basal metabolic rate (BMR) and *T*_b_ approaches ambient temperature (Barnes, 1989; Buck and Barnes, 2000). The suppression in BMR and *T*_b_ characterize the torpid state which is periodically interrupted by interbout arousals initiated by non-shivering thermogenesis (NST) *via* activation of BAT (Cannon and Nedergaard, 2004). While regulation of thermogenesis is important to sustain drastic changes in *T*_b_ and metabolic rate during the hibernation season, the seasonal regulation of thermogenesis is not fully understood. Previous work showed that AGS have a different seasonal response to pharmacologically induced torpor (Jinka et al., 2011). ^6^N-cyclohexyladenosine (CHA), an adenosine A_1_ receptor agonist, induces similar decrease in *T*_b_ as seen in hibernation during winter, but not in summer underlining an endogenous seasonal modulation in thermogenesis independent of environmental factors such as light cycle and ambient temperature (Jinka et al., 2011). AGS are robust hibernators where the hibernation phenotype is strongly regulated by season. In hibernators, BAT is the main site of NST (Ballinger and Andrews, 2018). BAT thermogenesis is regulated synergistically by the sympathetic nervous system (SNS) and thyroid hormones (TH; Ortiga-Carvalho et al., 2016). Hypothalamic–pituitary–thyroid (HPT) axis regulates TH production, increasing circulating TH to enhance thermogenic response when BAT is activated by the SNS (Silva and Larsen, 1983). Previous work in hibernation showed changes in circulating TH during torpor (Demeneix and Henderson, 1978; Nevretdinova and Shvareva, 1987; Magnus and Henderson, 1988) and thyroid morphology (Nunez and Becker, 1970; Krupp et al., 1977) in independent studies in different species. Here, we asked if seasonal modification of the HPT axis activation in AGS across season and in response to CHA-induced cooling could explain the seasonal response to CHA. As BAT thermogenesis is also regulated by sympathetic premotor neurons in the raphe pallidus (rPA; Nakamura et al., 2004), we also describe the SNS through the rPA neuronal activity across season and in response to CHA.

## Materials and Methods

All procedures were approved by Institutional Animal Care and use Committee (IACUC) at the University of Alaska Fairbanks and conducted in accordance with the Guide for the Care and Use of Laboratory Animals (8th edition). The objective of this study was to define the role of the HPT axis in seasonal hibernation and in the seasonal modulation of CHA-induced cooling. We treated animals with CHA or vehicle in both summer and winter seasons. Because vehicle did not trigger changes in *T*_b_ in either winter or summer seasons, we included vehicle-treated animals in our analysis of seasonal changes. We used animals treated with vehicle in summer as Summer euthermic (Sum.VEH) and animals treated with vehicle in winter as interbout euthermic (ibe), also referred to as Winter euthermic (Win.VEH). For seasonal comparisons, we added samples from naïve animals at two additional time points, Fall (pre-hibernation) and Torpor.

### Animals

**FIGURE 1:**
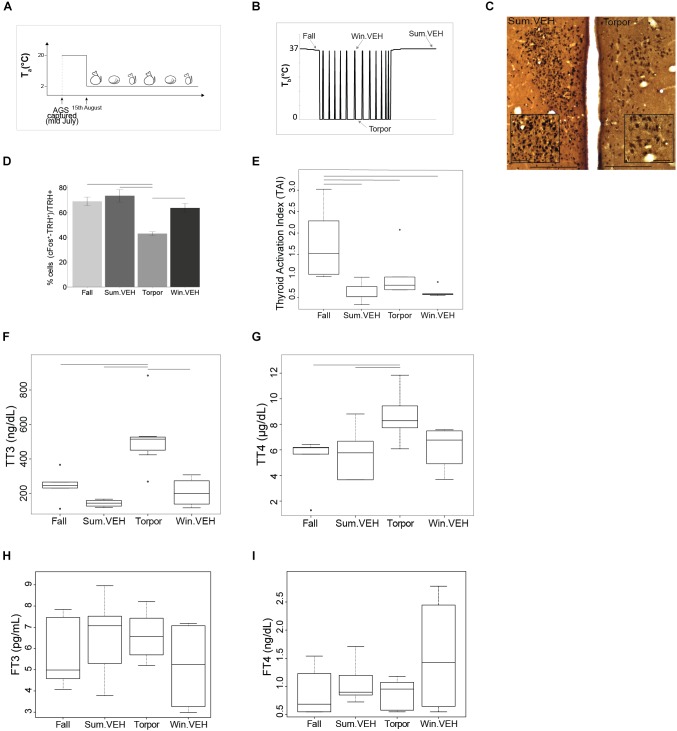
HPT-axis is active in summer and winter euthermia, but not in torpor. All AGS in the study were maintained under the constant environmental conditions (4L:20D and *T*_a_ = 2°C) after capture as shown in **(A)**, AGS maintain seasonal rhythm under the set constant environmental conditions. Results from samples collected at times shown in **(B)**, illustrate that TRH activation in the PVN is lower in Torpor **(D)**. Photomicrographs of double stained brain sections in **(C)**, TRH neurons (brown) and cFos (black) in the PVN are shown in Sum.VEH, as representative of the higher activation group, and in torpor; the insert in PVN images show higher magnification area, scale bar 200 μm (lower magnification), scale bar 50 μm (higher magnification). Thyroid activity is significantly higher during the Fall **(E)** and concentration of TT3 and TT4 is higher in Torpor **(F,G)**. No changes have been detected in FT3 or FT4 **(H,I)**. Symbols in **(E–G)** represent outliers. Horizontal bars represent differences between groups, *p* < 0.05, *t*-test in **(D,G)** and Wilcoxon’s rank-sum test in **(E,F)**, *n* = 4–7.

Arctic Ground Squirrels (AGS, *Urocitellus parryii*) were captured in the Brooks Range (68°07′46″N 149°28′33″W) under permit by Alaska Department of Fish and Game. Beginning August 15th, the year of capture, we housed AGS, male and female, individually at constant environmental conditions at an ambient temperature (*T*_a_) of 2°C and a photoperiod of 4L:20D until the end of the study to avoid any confounding results due to change in light cycle and *T*_a_ (Figure 1A). We provided approximately 47 g Mazuri rodent chow daily and water *ad libitum* although hibernating AGS do not drink or eat. Juveniles (one year old) were preferred for the study, adults were used when larger sample size was required.

### Seasonal Changes in the HPT-Axis

**FIGURE 2:**
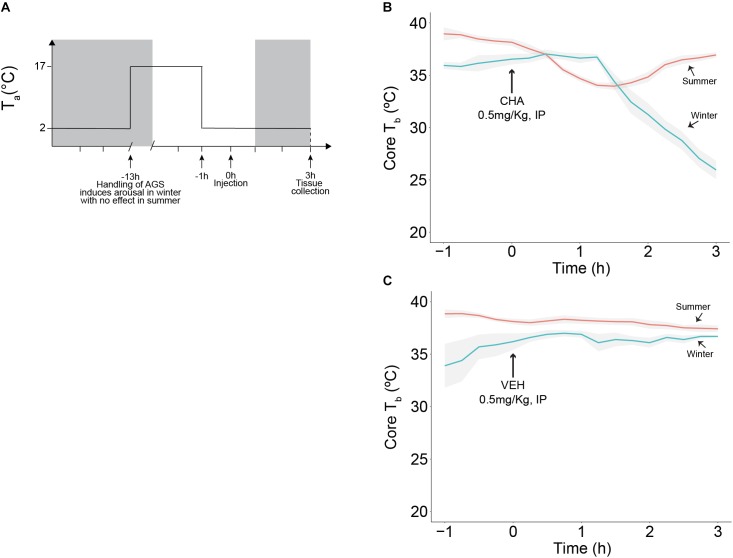
CHA, adenosine A_1_ receptor agonist, promotes onset of hibernation in a seasonally dependent manner. Experimental design is illustrated in **(A)**, treatment (CHA or VEH) was administered at time 0 and tissue collection was performed 3 h after injection, 2 h into the dark cycle (dark cycle is represented by gray area). CHA promotes the onset of hibernation in Winter AGS. Summer AGS respond to CHA briefly and restore the initial *T*_b_ value **(B)**. Vehicle does not affect *T*_b_ **(C)**. Data shown as mean ± SEM, *n* = 4–7. Arrow at 0 h indicates time of injection.

To identify seasonal changes at the level of the HPT axis, we collected tissues at different time points defined as Fall, Winter euthermic (Win.VEH), Torpor, and Summer (Sum.VEH) (Figure 1B). Torpor was monitored daily using the shaving added technique (Jameson, 1964). The animal was defined torpid if wood shaving placed on the back remained undisturbed 1-day later. The presence of eight torpor bouts defined a winter phenotype that we referred to as a winter season. Summer phenotype (referred to as summer season) was characterized by at least 60 consecutive awake days following the end of the hibernation season. The fall transition state was characterized by a slight decrease in euthermic *T*_b_. Rectal *T*_b_ was measured in all animals before intracardial perfusion with a Digital Microprocessor Thermometer (model HH21 OMEGA Engineering, INC., Stamford, CT, United States). Table 1 reports details on each seasonal phenotype. We collected all the animals at the same time of day, 2 h into the dark cycle. Fall and torpid AGS were naïve and torpid AGS were collected between the first and fourth day of the torpor bout, consistent with the time of induced-arousal of winter AGS. Win.VEH and Sum.VEH were collected following the procedure illustrated in Figure 2A and described as follows. Brain, thyroid tissue, and blood samples were collected as described below.

**Table 1:** Characteristics of each seasonal phenotype.

Seasonal phenotype	Sample size	Torpor bouts	Body weight (g)	Rectal *T*_b_ (°C)
Fall	7	0	699 ± 64^b,c^	35.4 ± 0.3^a^
Torpor	4	≥ 8	594 ± 35^c^	2.9 ± 0.3^c^
Ibe (Winter vehicle)	5	≥ 8	486 ± 28^a^	36.1 ± 0.3^a^
Summer (Summer vehicle)	7	0	584 ± 33^a,c^	37.0 ± 0.2^b^

*Data shown ± SEM, rectal T_b_ was measured prior to tissue collection. Groups annotated with different letters ^(a,b,c)^ are significantly different from groups annotated with the same letters p < 0.05, Wilcoxon’s rank-sum test.*

### Changes in the HPT-Axis After CHA-Induced Cooling

**FIGURE 3:**
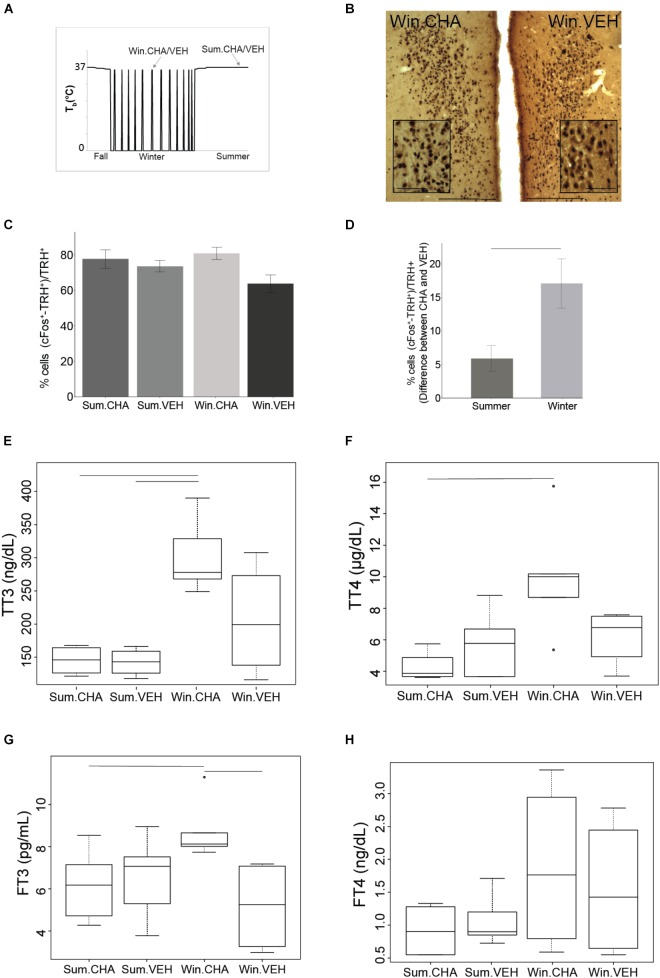
CHA activates HPT axis more in winter than in summer. Results from samples collected at times shown in **(A)**. Photomicrographs of double stained brain sections in **(B)**, TRH neurons (brown) and cFos (black) in the PVN are shown in VEH and CHA animal, the insert in PVN images show higher magnification area, scale bar 200 μm (lower magnification), scale bar 50 μm (higher magnification). TRH neurons are activated after CHA with no effect of season **(C)**. Significantly higher activation of TRH neurons after CHA in Winter compared to Summer was evident when AGS were matched for age and gender. Data shown are mean ± SEM of differences between CHA and vehicle-treated pairs matched for age and gender **(D)**. CHA increases TT3 and TT4 more in Winter than in Summer **(E,F)**, FT3 increases after CHA in Winter **(G)**. FT4 does not change after CHA **(H)**. Symbols in **(F,G)** represent outliers. Horizontal bars represent differences between groups, *p* < 0.05, Wilcoxon’s rank-sum test in **(E**,**F)**, *t*-test in **(C,D,G)**, *n* = 4–7.

AGS were implanted with iButton, (Maxim integrated, San Jose, CA, United States) abdominal temperature data loggers. Anesthesia was induced with 5% isoflurane and maintained at 3% mixed with 100% medical grade oxygen delivered at a flow rate of 1.5 L/min. During surgery *T*_b_ was maintained *via* a water circulating heating pad. Under aseptic conditions, the iButton was inserted intraperitoneally *via* a midline incision through linea alba and sealed with three layers of sutures. Buprenorphine (1.0 mg/kg, sc) slow release formulation was administered as analgesic and AGS were allowed at least 1 day of post-operative recovery in a warm room (17°C, 4L:20D) before being returned to home cage conditions (*T*_a_ of 2°C and 4L:20D). We induced arousal in winter AGS between the first and fourth day of the torpor bout and we moved the aroused animals to a warm room (17°C, 4L:20D) to maintain arousal state and to delay the occurrence of the following torpor bout. Animals displaying the summer phenotype were also moved to a warm room overnight (17°C, 4L:20D), for consistency in the procedure. During the overnight period, (approximately 12 h) food was withheld, and water was available *ad libitum*. In the morning of the experiment, AGS were moved to the environmental chamber (2°C, 4L:20D) 1 h before injection to acquire baseline data. Vehicle (2.5% hydroxypropyl-β-cyclodextrin) or CHA (0.5mg/kg, IP) were injected and animals were monitored for 3 h. At 3 h after injection blood was collected *via* cardiac puncture and AGS were perfused intracardially prior to tissue collection. A schematic of the experimental designed is illustrated in Figure 2A and the related time points for tissue collection are shown in Figure 3A.

### Drugs

A stock solution of CHA (10 mg/mL, Sigma-Aldrich, Saint Louis, MO, United States) was dissolved in 25% (w/v) hydroxypropyl-β-cyclodextrin (Tokyo Chemical Industry CO., Tokyo, Japan) in sterile water. On the week of the experiment, stock solution of CHA was diluted to 0.5 mg/mL in sterile normal saline (0.9% NaCl). Diluted CHA was used within 1 month. Solutions for injection were sterilized by 0.2 μm filtration (Acrodisc syringe filter; Sigma-Aldrich, Saint Louis, MO, united States).

### Brain Tissue Processing

At 3 h after injection, AGS were anesthetized with 5% isoflurane and maintained at 3% mixed with 100% medical grade oxygen delivered at a flow rate of 1.5 L/min. Blood was sampled by cardiac puncture and AGS were intracardially perfused first with 0.9% NaCl for 5 min and then with 4% PFA in 0.1 M PB buffer pH 7.4 at a flow rate of 79.5 mL/min with a 18 Ga needle through the left ventricle after the descending aorta was clamped to obtain a more efficient perfusion of the brain. Brains were removed and post fixed in 4% PFA overnight. Brains were blocked in 3 parts to allow better penetration of sucrose into the tissue. A gradient of sucrose solutions (5, 10, 15, 20, and 30 % w/v) was made in 0.1M PB buffer pH 7.4 to cryoprotect the tissue. Brains were maintained in each sucrose solution for up to 3 days and in 30% sucrose until brains sank. Brains covered with Tissue-Tek O.C.T. matrix (Electron Microscopy Sciences, Hatfield, PA, United States) were rapidly frozen in a bath of n-Hexane 95% cooled with dry ice to a temperature of -45°C. Coronal sections (40 μm) were cut with a cryostat (CM1850, Leica Biosystems, Buffalo Grove, IL, United States).

### Immunohistochemistry

Free floating sections were washed using PBS pH 7.2, blocked with normal goat serum (Vector Laboratory, Burlingame, CA, United States) for 2 h at 4°C and labeled with mouse anti-cFos (1:20,000, Santa Cruz Biotechnology, Dallas, TX, United States) in PBS-0.2% TritonX-100 for 48 h, followed by incubation with biotinylated secondary goat anti-mouse antibody 1:600 (Vector Laboratory, Burlingame, CA, United States) in PBS-0.4% TritonX-100. After incubation in avidin-biotin-peroxidase (Vectastain ABC kit, Vector Laboratory, Burlingame, CA, United States), sections were treated with 0.05% DAB (3′,3-diaminobenzidine tetrahydrochloride, Sigma-Aldrich Corp. St. Louis, MO, United States) with 1% ammonium nickel sulfate in sodium acetate 0.175M and 0.1% H_2_O_2_. For double stain procedures, slices were then incubated with the second primary antibody, rabbit anti-TRH (1:10,000, gift from Eva Redei Northwestern University) or sheep anti-tryptophan hydroxylase (TPH) (1:2,000, AB1541, Millipore) in PBS-0.2% TritonX-100 overnight, followed by incubation with biotinylated secondary goat anti-rabbit or goat anti-sheep 1:600 (Vector Laboratory, Burlingame, CA, United States) in PBS-0.4% TritonX-100. After incubation with ABC, sections were treated with 0.05% DAB in PBS and 0.1% H_2_O_2_. Sections were mounted on Superfrost Plus slides (VWR, Radnor, PA, United States) with cytoseal mounting media (Thermo Fisher Scientific, Waltham, MA, United Sates) or Permount (Thermo Fisher Scientific, Waltham, MA, United States). Control slices, in which primary antibody was omitted and replaced by PBS, were run in every experiment under the same conditions.

### Image Analysis

Images were analyzed using Metamorph software (version 7.8.8.0, Molecular Devices, San Jose, CA, United States) with a Nikon Eclipse TE2000-U inverted microscope equipped with a CCD camera (Cool Snap HQ2, Photometrics, Tucson, AZ, United States) for double stain analysis and with Nikon Eclipse 80i equipped with a digital camera (Micropublisher 3.3RTV, Qimaging, BC, Canada) for single stain analysis. A blind code was assigned to each animal to prevent any bias from the observer. For TRH analysis each hemisphere was counted individually in three consecutive slices for each AGS. The mean of six slices, three from each hemisphere per AGS, was used for subsequent statistical analysis. The count was done manually under a 20X objective using the manual count function in Metamorph to prevent double counting. Double stain was defined as brown DAB neurons with a distinct round dark black nucleus. All TRH positive neurons and the double stained neurons were counted in the paraventricular hypothalamic nucleus (PVN). The PVN was identified by reference to the Paxinos and Watson Rat Atlas Fourth Edition (bregma -1.40 to -1.60 mm). The percentage of activated TRH neurons was defined as the ratio between double stained neurons and the number of TRH positive neurons. We identified the rPA by Nissl stain with reference to the Paxinos and Watson Rat Atlas Fourth Edition (bregma -11.30 to -11.80 mm) and TPH staining. We defined a region of interest (ROI) of 0.05 mm^2^ to include the entire rPA. cFos+ neurons were counted manually in the ROI in four consecutive slices under 10X objective in Metamorph.

### Thyroid Tissue Processing and Image Analysis

After perfusion each thyroid lobe was isolated and stored in 70% ethanol until tissue processing. Paraffin sections (4 μm) were stained with hematoxylin and eosin (Carson, 1997). The first slice was collected when the tissue was exposed and the next four slices were collected at 50–80 μm intervals. Histological analyses were performed in bright field using the Nikon Eclipse 80i upright microscope under 40X objective. Stereological analyses were performed using the point-counting method. A grid of points was placed on top of the image at final magnification of 400X. A total of 15 randomly selected fields of view were analyzed per each section for a total of 4 sections per animal. A total of 60 fields of view per animal were used to calculate the thyroid activation index (TAI). First the volume of the colloid and follicular epithelium (*V*_vph_ ) was calculated based on the following equation *V*_vph_ = *P*_ph_/*P*_tot_, where *P*_ph_ is number of points on a particular phase (colloid or epithelium) and *P*_tot_ is the total number of points of the grid (Weibel et al., 1966), TAI was calculated based on the epithelial (*V*_ve_) to colloid (*V*_vc_) volume density ratio TAI = *V*_ve_/*V*_vc_ (Kalisnik, 1972).

### TH Analysis

Blood was collected *via* cardiac puncture using a heparinized syringe. Plasma was separated by centrifugation at 3,000 ×*g* for 8 min at 4°C and then store at -80°C until use. TH were measured using micro high performance liquid chromatography-tandem mass spectrometry (LC-MS/MS) as previously described (Gu et al., 2007; Jonklaas et al., 2009; van Deventer et al., 2011; Masika et al., 2016).

For total thyroid hormones (TTH) analysis, 150 μL internal standards (IS) L-Thyroxine-[L-Tyr-d5] hydrochloride (T4-d_5_) and 3,3’,5-Triiodo-L-thyronine-^13^C_6_ (T3-^13^C_6_) were added to 200 μL plasma (thawed at RT) and mixed using a vortex for 30 s, then centrifuged for 10 min at 13,000 rpm. We added 500 μL 0.1M ammonium acetate in water to 200 μL supernatant and vortexed for 10 s before HPLC separation using a mobile phase A of 2% methanol with 0.01% formic acid and a mobile phase B of 98% methanol with 0.01% formic acid. For free thyroid hormones (FTH) analysis, 400 μL plasma was placed in a 30-kDa ultrafiltration device (Centrifree YM-30, Millipore) and centrifuged in an Eppendorf temperature-controlled centrifuge at 2,700 rpm for 40 min at 37°C. To 150 μL ultrafiltrate was added 250 μL IS. We added L-Thyroxine-^13^C_6_ (T4-^13^C_6_) and 3,3’,5-Triiodo-L-thyronine-^13^C_6_ (T3-^13^C_6_) as IS to each aliquot and mixed using a vortex for 30 s, then centrifuged for 10 min at 13,000 rpm. The supernatant, diluted with water, was separated *via* HPLC using mobile phase A of 2% methanol with 0.01% acetic acid and mobile phase B of 100% methanol with 0.01% acetic acid.

Each sample, prepared as described, was then injected onto an Agilent SB C-18 (2.1 mm 50 mm, 3.5 m ID) chromatographic column (injection volume was 200 μL for TTH analysis and 400 μL for FTH analysis). The HPLC system consisted of 3 Shimadzu LC-20AD pumps, a Shimadzu SIL-HTA autosampler, and a Shimadzu DGU-20A5 degasser. The procedure involved an online extraction step followed by activation of a built-in Valco switching valve and subsequent sample introduction into the mass spectrometer. After a 3-min wash with 20% (vol/vol) methanol in 0.01% acetic acid at a flow rate of 1.0 mL/min, the switching valve was activated and the analytes of interest were eluted from the column and introduced into the mass spectrometer with a water/methanol gradient. We used an API-5000 tandem mass spectrometer (Applied Biosystems/MDS Sciex) equipped with TurboIonSpray source (ionspray voltage 4200V), operated in the negative ionization multiple reaction monitoring (MRM) mode. The MRM transitions monitored for T4-d_5_, T3-^13^C_6_, T4, and T3 were 783/135, 658/124, 778/150, and 652/150, respectively in the TTH assay. The MRM transitions monitored for T4, T3, T4-^13^C_6_, and T3-^13^C_6_ were 776/127, 650/127, 782/127, and 656/127, respectively in the FTH assay. Data were acquired and processed by Analyst 1.4.1 software package. HPLC-grade methanol was from Fisher Scientific. The lower limits of detection were 0.55 ng/dL for Free T4 (FT4), 1.00 pg/mL for Free T3 (FT3), 0.25 μg/dL for T4, and 5.50 ng/dL for T3. Non-detectable values were assumed to be equal to the lower detection limit for statistical analysis. Intra assay variability reported as coefficient of variations ranged between 5.3 and 11.5% for FTH and 3.4–5.2% for TTH. The inter assay variability ranged between 9.4 and 12.6% for FTH and 4.8 and 6.2% for TTH.

### Statistical Analysis

Data are presented as mean ± SEM and a *p* < 0.05 was considered significant. Data analyses were performed using R (version 1.1.423). Groups with both season and treatment as independent variables were tested with a two-way ANOVA. To allow for a two-way ANOVA in the cell count analysis, data were normalized by transforming to the square root. ANOVA was followed by independent samples *t*-test. One-way ANOVA was used when season was the only independent variable and data were normally distributed. Kruskal Wallis and Wilcoxon signed-rank tests were used when data were not normally distributed. Groups were randomly matched by sex and age where indicated.

## Results

Body weight and rectal *T*_b_ of animals collected in Fall, Winter, Torpor, and Summer were consistent with the seasonal phenotype of hibernation (Figure 1B and Table 1).

### Seasonal Changes in the HPT-Axis

To asses seasonal changes in the HPT axis we defined HPT axis activation based on up-regulation at any point of the axis beginning with activation of TRH+ neurons in the PVN, and including TAI (Kalisnik, 1972) and circulating total and FTH. We found that TRH neurons were least active during Torpor [*F*(3,16) = 11.13, *p* < 0.001 one-way ANOVA, followed by *t*-test] compared to the three different euthermic states (Figures 1C,D). Suppressed activity in TRH neurons is consistent with reduced thermogenesis and metabolic rate during torpor. Surprisingly, TAI in Torpor was not lower compared to Summer or Winter euthermia. Moreover, TAI was significantly higher in Fall AGS compared to the other three states [*H*(3) = 13.48, *p* < 0.01, Kruskal Wallis test, followed by Wilcoxon’s rank-sum test]. In fact, TAI trended toward higher activation in Torpor compared to Winter euthermic animals (*p* = 0.051, Wilcoxon’s rank-sum test, Figure 1E). Thus, thyroid activity is maintained between Summer, Winter, and Torpor and is significantly elevated in the Fall AGS.

Next, we measured the plasma levels of TH. Both total l-Thyroxine (TT4) and total 3,3***′***,5-Triiodo-L-thyronine (TT3) are highest during torpor (Figures 1F,G). Plasma concentration of TT4 is significantly higher in Torpor compared to Summer euthermic and Fall [*F*(3,18) = 3.74, *p* < 0.05 one-way ANOVA, followed by *t*-test]. As with TAI, TT4 in torpor trends toward higher levels compared to Winter euthermic (*p* = 0.07, *t*-test, Figure 1G). Likewise, plasma concentration of TT3 is significantly higher in Torpor compared to the three different euthermic states [H(3) = 14.39 *p* < 0.01 Kruskal Wallis test, followed by Wilcoxon’s rank-sum test, Figure 1F]. Consistent with higher concentration of TTH in torpor, we found inverse correlations between TT3, TT4 and T_b_ (Supplementary Figures S3A,B). Neither FT3 nor FT4 changed significantly across season [H(3) = 1.86 *p*-value > 0.05 Kr***u***skal Wallis test, FT3; [*F*(3,18) = 0.85, *p* > 0.05 one-way ANOVA, FT4; Figures 1H,I]. [H(3) = 1.86 *p*-value > 0.05 Kruskal Wallis test, FT3; *F*(3,18) = 0.85, *p* > 0.05 one-way ANOVA, FT4; Figures 1H,I].

We did not find any significant change in plasma levels of reverse triiodothyronine (rT3) the pharmacologically inactive hormone between seasons (Supplementary Figure S1A), however the ratio between rT3 and TT3 was significant lower in torpor compared to Summer and Winter euthermic animals, suggesting a downregulation of the HPT axis in torpor (*p* < 0.05, Supplementary Figure S2A).

### CHA-Induced Cooling

Except for the fall transition period, our data support consistent HPT axis activation during euthermia across seasons. We next asked if HPT axis activation changes during onset of torpor mimicked by CHA-induced cooling. Consistent with previous observations, CHA administered to Winter AGS gradually decreased *T*_b_ to 26.0 ± 0.9°C after 3 h. By contrast, CHA in Summer AGS produced a rapid, but transient decrease in *T*_b_ that returned to euthermic values 36.9 ± 1.9°C within 3 h [*H*(17) = 17.8, *p* < 0.001, Kruskal Wallis test, Figure 2B]. Vehicle treatment did not change *T*_b_ [*H*(16) = 8.4, *p* = 0.9, Kruskal Wallis test, Figure 2C]. Also consistent with previous observations, resting *T*_b_, reported as mean of *T*_b_ measured in the 1 h prior injection, was lower in Winter AGS (36.4 ± 0.3°C) compared to Summer AGS (38.6 ± 0.2°C, *p* < 0.001, *t*-test). Such seasonal changes provide evidence of a seasonal decrease in thermogenesis as seen by significantly lowering euthermic *T*_b_ reported in Table 1.

### Changes in the HPT-Axis After CHA-Induced Cooling

At the level of TRH neuronal activation, CHA activated the HPT axis [percent of cFos+ and TRH+ over TRH+ after CHA (79 ± 3%) compared to vehicle (69 ± 3%), *F*(1,17) = 5.562, *p* = 0.03, two-way ANOVA, Figures 3B,C] but the interaction with season was not significant. After the two-way ANOVA failed to show an interaction with season we minimized inter-animal variation by matching pairs of animals by sex and age; sex and age are known to influence TH concentrations (Suzuki et al., 2012). In the analysis of TRH neuronal activation as the difference between matched pairs, we found a significant influence of season. Matched pairs showed that CHA activated more TRH neurons in Winter than in Summer (Figure 3D). Greater activation supports an increased thermogenic capacity in winter. Looking at the next level of HPT axis activation TAI was stable across CHA and vehicle treatment groups [*F*(1,15) = 0.001,*p* = 0.98, two-way ANOVA]. Although TAI did not change, TTH increased in parallel to TRH neuronal activation after CHA in Winter compared to CHA in Summer. Following CHA administration, both concentrations of TT4 and TT3 are higher in winter AGS compared to summer (*p* < 0.05, Wilcoxon’s rank-sum test, Figures 3E,F). Consistent with increased HPT axis activation after CHA in Winter, we found greater increases in FT3 after CHA in Winter than after CHA in Summer [*F*(1,15) = 5.34, *p* < 0.05 two-way ANOVA, Figure 3G]. FT4, however, was not affected differently by CHA in Summer and Winter (*p* > 0.05 Wilcoxon’s rank-sum test, Figure 3H). We did not find any significant change in the plasma levels of rT3 and in the ratio between rT3 and TT3 between the treatment groups (Supplementary Figures S1B, S2B). The greater HPT axis activation in response to CHA in Winter AGS compared to Summer is consistent with greater thermogenic capacity during the winter season.

### Seasonal Changes in the SNS and in CHA-Induced Cooling

**FIGURE 4:**
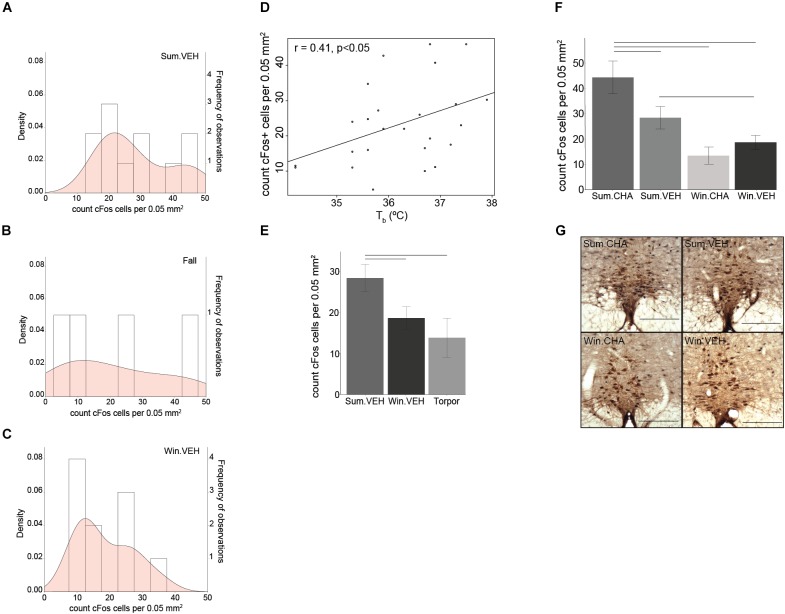
Sympathetic tone decreases in winter. The density (probability distribution) of active neurons in the rPA is wider in Fall **(B)** compared to Summer **(A)** and Winter **(C)**. A seasonal decrease in euthermic *T*_b_ correlates with a seasonal decrease in rPA neuronal activity **(D)**. rPA activation is higher in Summer euthermia compared to Torpor and Winter euthermia **(E)** and significantly increases after CHA in Summer, but not in Winter **(F)**. Photomicrographs of double stained brain sections in **(G)**, TPH neurons (brown) used to identify the rPA and cFos (black) are shown in the 4 treatment groups (Sum.CHA, Sum.VEH, Win.CHA, and Win.VEH), scale bar 200 μm. Horizontal bars represent differences between groups, *p* < 0.05, *t*-test in **(E,F)**, *n* = 4–11.

**FIGURE 5:**
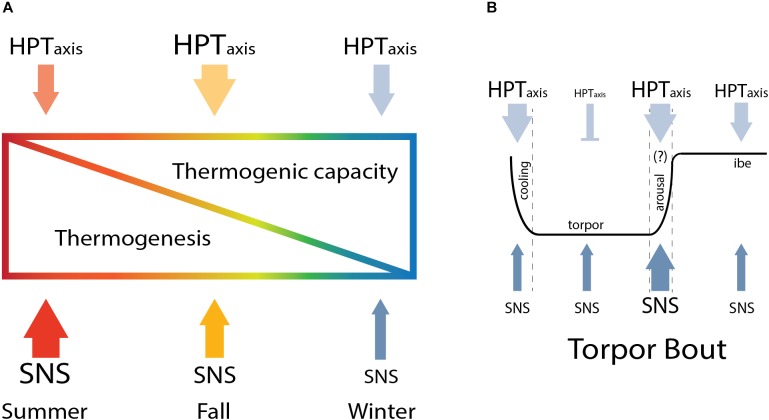
Seasonal modulation of thermoregulation in hibernation. The HPT axis and SNS change across season to increase thermogenic capacity in winter and gradually reduce thermogenesis from summer to winter **(A)** until arousal, when the higher thermogenic capacity is critical to sustain thermogenesis needed to rewarm from torpor **(B)**.

In hibernating rodents, BAT activation is a significant source of thermogenesis, and BAT mass seasonally increases to sustain periodic arousals. Because BAT thermogenesis is regulated by the SNS *via* activation of sympathetic premotor neurons in the rPA, we next asked if changes in the rPA are associated with seasonal changes in thermogenesis. rPA activation, measured as the total number of cFos+ cells, decreases from Summer to Winter. Density plots visualize the distribution of the number of activated cells in the rPA; we chose a density plot, because it defines the distribution shape better than a frequency histogram (also shown) when the number of bins is small. Distribution of cFos+ cells in the rPA shows a transition between the highest activity in Summer (Figure 4A) to the lowest activity in Winter (Figure 4C) with a broader distribution of rPA neuronal activity during the Fall transition season (Figure 4B). The adjustment in rPA neuronal activity correlates positively with *T*_b_ (*r* = 0.41, *p* < 0.05, Figure 4D). Due to the broader distribution of rPA activation in the fall, the fall group was omitted from the subsequent analysis and ANOVA was performed between Torpor, Winter, and Summer groups. We found significantly higher activation in Summer compared to Winter and Torpor [*F*(2,22) = 4.16, *p* < 0.05, one-way ANOVA, followed by *t*-test, Figure 4E]. Compared to Vehicle, CHA induced rPA activation in Summer, but not in Winter [*F*(2,35) = 6.6, *p* < 0.01, two-way ANOVA, followed by *t*-test, Figures 4F,G]. Although thermogenic capacity is increased in winter, these results suggest that a decrease in sympathetic premotor neuronal activity attenuates thermogenesis during the winter season (Figure 5).

## Discussion

Here, we describe results that support a model in which thermogenic capacity increases in the hibernation season despite a decrease in sympathetic premotor neuronal activity and sympathetic response to cooling which is attenuated until interbout arousal. Thermogenesis is regulated within the rPA to gate SNS activation of shivering and NST (Tan and Knight, 2018). BAT thermogenesis, the primary form of NST in rodents, is regulated by both SNS and TH. Thermogenic capacity increases in winter as we find a greater response of HPT axis to CHA-induced cooling in winter compared to summer. HPT axis response is evident from higher TRH neuronal activation within the PVN and the increase in circulating FT3 and TTH during CHA-induced cooling. Although thermogenic capacity increases, overall sympathetic premotor neuronal activity needed to stimulate thermogenesis decreases during the winter season. We interpret the data as a decrease in sympathetic activity based on a lower euthermic *T*_b_ during winter compared to summer and on reduced activation of rPA neurons during winter compared to summer euthermia. We also see a decreased sympathetic response to cooling evident from lower rPA neuronal activation following CHA in winter compared to summer. Our data suggest that SNS activity changes during the fall season indicated by a wider distribution of the number of activated rPA neurons in fall compared to summer or winter. Increased TAI further supports modification of the HPT axis during the fall transition. Enhancement of HPT axis capacity during the fall transition establishes higher thermogenic capacity necessary to support periodic arousals. The higher thermogenic capacity is needed to rewarm from an average of 2.9–36°C in about 10 h during periodic arousal (Karpovich et al., 2009) as well as a protective mechanism to defend *T*_b_ when the thermal gradient between *T*_b_ and *T*_a_ increases (Richter et al., 2015). On the other hand, AGS suppress thermogenesis as seen in the rPA to allow the onset of hibernation, overwriting the thermogenic response at the level of the HPT axis in CHA-induced cooling. Our model describes how hibernators finely regulate thermogenesis across seasons. A lower thermogenesis in winter allows the onset of hibernation and the parallel increase in thermogenic capacity is crucial to enhance thermogenesis when needed.

Other published literature supports our model. Thermogenic capacity measured as maximum NST in Richardson’s ground squirrel is higher in winter animals (Abbotts and Wang, 1980). Seasonal changes in BAT also demonstrate increased thermogenic capacity in winter. BAT mass and BAT UCP1 mRNA increases in winter compared to summer hibernators (Kitao and Hashimoto, 2011; Ballinger and Andrews, 2018). Consistent with our model describing a period of fall transition in thermogenic capacity, a recent study showed that BAT mass increases during fall (Ballinger et al., 2016) and changes in thyroid morphology, similar to what we observed, occur in marmots and bats during the pre-hibernation season (Nunez and Becker, 1970; Krupp et al., 1977). Early thyroidectomy studies in hibernators underline the importance of this gland for hibernation and the role the thyroid plays to enhance thermogenic capacity to sustain periodic arousals. Thyroidectomy performed after cold exposure during the fall, does not affect hibernation as torpor bouts persist (Henderson et al., 1981), however, when thyroidectomy is performed in spring lethal hypothermia occurs (Canguilhem, 1972). Other studies in AGS and 13-lined ground squirrels illustrate a gradual decrease in *T*_b_ before the onset of hibernation (Barnes, 1989; Russell et al., 2010; Sheriff et al., 2012; Olson et al., 2013) consistent with our results. As thermogenic capacity increases in fall, basal thermogenesis decreases as seen by the modulation in *T*_b_.

In this study, we minimized the influence of feeding and gastric motility on rPA neuronal activity (Jacobs et al., 2002) by withholding food for approximately 12 h before the experiment. The positive association between *T*_b_ and the number of cFos+ cells within the rPA suggests that rPA neuronal activity is related to thermogenesis. Nonetheless, the influence of other autonomic nervous system responses on rPA cannot be ruled out and deserves further study.

The increase in TTH during torpor is also seen in 13-lined ground squirrel and hamster using RIA to detect TTH (Demeneix and Henderson, 1978; Nevretdinova and Shvareva, 1987; Magnus and Henderson, 1988). We also note that our TTH values, determined by LC-MS/MS are consistent with values measured by RIA in studies cited above. Increased TTH in torpor is consistent between species, however, changes in FTH values are not consistent. Our results are in agreement with previous study in Djungarian hamsters where FT4 and FT3 do not change during mid-winter torpor. Djungarian hamsters, however, do show an increase in FT3 during late season torpor in preparation for the reproductive phase (Seidel et al., 1987). In Richardson’s ground squirrel results are contradictory. One study reports an increase in FT4 during torpor compared to arousal (Demeneix and Henderson, 1978) while another shows the opposite with FTH decreasing in torpor compared to arousal (Magnus and Henderson, 1988). Differences in results may be due to species differences and to methods used to separate bound and free hormones. We used the ultrafiltration method instead of the classical equilibrium dialysis (ED) to separate FTH from bound TH. Ultrafiltration is becoming the preferred alternative method to ED in clinical settings (Soldin and Soldin, 2011). Clinical works show a better correlation with thyroid stimulating hormones when FTH are measured using MS instead of immunoassay, suggesting higher accuracy and precision of LC/MS-MS in quantifying FTH concentration compared to ED-RIA (van Deventer et al., 2011).

Our analysis of the HPT axis is limited by a lack of deiodinase levels in BAT. Deiodinase regulates intracellular TH levels, in particular type 2 iodothyroinine deiodinase (Dio2) converts T4 to T3. Although we did not asses the Dio2 levels in AGS, previous studies in hamster reported seasonal changes in Dio2 levels in hypothalamus during short photoperiod (Herwig et al., 2009) and in BAT during torpor compared to normothermia (Bank et al., 2015). These studies suggest that a low level of T3, as a consequence of lower Dio2 expression, promotes torpor. Thus, while we show higher TTH and no change in FTH during torpor in AGS, the intercellular level of T3 may be decreased by lower Dio2 expression that we were unable to measure. Together, lower Dio2 expression and lower sympathetic premotor neuronal activity may contribute to decreased thermogenesis during torpor.

We are aware of technical limitations in our study such as the use of vehicle-treated animals for assessment of seasonal changes and the small sample size of some of the groups. It is unlikely that vehicle treatment confounds our interpretation since vehicle injection had no effect on *T*_b_. Moreover, TTH concentration previously reported in AGS (Nevretdinova and Shvareva, 1987) was in the same range as values reported in vehicle-treated animals in our study, suggesting a minimal to null effect of handling on the parameters measured in the study. The sample size used depended on the availability of wild caught animals which is limited by the seasonal nature of hibernation and reproduction. Nonetheless, the sample size was sufficient to develop a model that can be further validated with future study.

The use of cFos immunoreactivity to detect neuronal changes across season may be considered a methodological limitation as cFos expression is often used to investigate acute challenges. However, cFos is also expressed during spontaneous waking and under basal conditions (Cirelli and Tononi, 2000; Kovács, 2008), making cFos a reliable marker to investigate physiological states as sleep–wake cycle (Cirelli and Tononi, 2000), daily rhythm (Ikeno et al., 2017) hibernation (Bratincsak et al., 2007), and seasonal changes (Mishra et al., 2018). Our data does not show any change in cFos activation in the rPA between winter euthermic (Win.VEH) and torpor, which is consistent with previous work showing constant cFos mRNA expression within the rPA during the hibernation season in 13-lined ground squirrels (Bratincsak et al., 2007).

In conclusion, we report changes in HPT axis activation that is associated with increased thermogenic capacity in winter. We also report lower rPA neuronal activity in the winter season and in response to CHA-induced cooling. We interpret these findings in the context of a model whereby increased thermogenic capacity in winter supports arousal. Meanwhile, attenuated sympathetic premotor neuronal activity promotes the onset of torpor.

This work is significant as an increase in thermogenic capacity in winter compared to summer also occurs in humans. In healthy subjects, higher thermogenic capacity is associated with higher BAT activation after cold exposure during winter season (Cohade et al., 2003; Saito et al., 2009; Ouellet et al., 2011; Senn et al., 2018) as well as an increase in BAT mass in winter (Ouellet et al., 2011). Furthermore, our results show a gradual decrease in thermogenesis from summer to winter during what is called physiological obesity in hibernation. The decrease in thermogenesis resembles human obesity. In obese individuals, BAT thermogenesis is impaired (Ooijen et al., 2006; Wijers et al., 2010; Orava et al., 2013; Loh et al., 2017) showing a blunted response to cold exposure compared to lean subjects. Our model combines a seasonal increase in thermogenic capacity (independent from photoperiod and *T*_a_) with a decrease in thermogenesis and further study may lead to the understanding of endogenous thermoregulatory mechanisms to use as therapeutic targets.

## Author Contributions

CF and KD designed the study and interpreted the data. CF and MJ performed the experiments. SS performed the hormone analysis. CF analyzed the data. CF and KD wrote the paper. All authors contributed to the revision of the manuscript and approved the final draft.

## Conflict of Interest Statement

KD has a financial interest in Be Cool Pharmaceutics. The remaining authors declare that the research was conducted in the absence of any commercial or financial relationships that could be construed as a potential conflict of interest.
