# Supplementary material for: The Raphe Pallidus and the Hypothalamic-Pituitary-Thyroid Axis Gate Seasonal Changes in Thermoregulation in the Hibernating Arctic Ground Squirrel (Urocitellus parryii)
Source: Front Physiol. 2018 Dec 12;9:1747. doi: 10.3389/fphys.2018.01747 (PMC6299024; doi:10.3389/fphys.2018.01747)
Supplement: Supplementary file 1 [file Table_1.DOCX]

Supplementary Material

The raphe pallidus and the hypothalamic-pituitary-thyroid axis gate seasonal changes in thermoregulation in the hibernating Arctic Ground Squirrel (Urociltellus parryii)

**Frare C., Jenkins M.E., Soldin S.J., Drew K.L. ***

*** Correspondence:** Kelly Drew kdrew@alaska.edu

# Supplementary Figures

**Supplementary Figure 1.** Level of reverse triiodothyronine (rT3) pharmacologically inactive hormone**.**  rT3 is considered a metabolically inactive thyroid hormone produced by the deiodination of T4 to rT3 catalyzed by type 3 iodothyronine deiodinase (Dio3), even though type 1 iodothyronine deiodinase (Dio1) can perform the conversion. The constant level across seasons (**A**) and between treatments (**B**) may be explained by a lack of significant alterations in the metabolism of circulating thyroid hormones. In eight of 31 AGS (26 %) the rT3 concentration was below the limit of detection. Wilcoxon’s rank-sum test in **A** and **B**, n=4 to 7.

**Supplementary Figure 2.** During torpor HPT axis is downregulated. The ratio between rT3 and TT3 is lower in torpor compared to winter euthermic and summer euthermic. In Fall the ratio is lower than summer euthermic but does not differ from winter euthermic, in agreement with Fall being a transition state where thermogenesis is modulated before hibernation starts (**A**). We did not detect any change in the ratio between treatments group (**B**). Horizontal bars represent differences between groups p<0.05, Wilcoxon’s rank-sum test in **A** and **B**, n=4 to 7.

**Supplementary Figure 3.** Higher concentration of TT3 and TT4 are associated with low T_b_ consistent with higher circulating levels at minimum T_b_ during torpor. We described an inverse correlations between TT3, TT4 and T_b_ using Pearson’s correlation when data were normally distributed and Spearman’s correlation when data were not normally distributed (for TT3, r_s_= -0.74, p <0.001, SI Fig. **3A**, for TT4 r_s_= -0.72, p <0.001, SI Fig. **3B**), n=4 to 7.

**
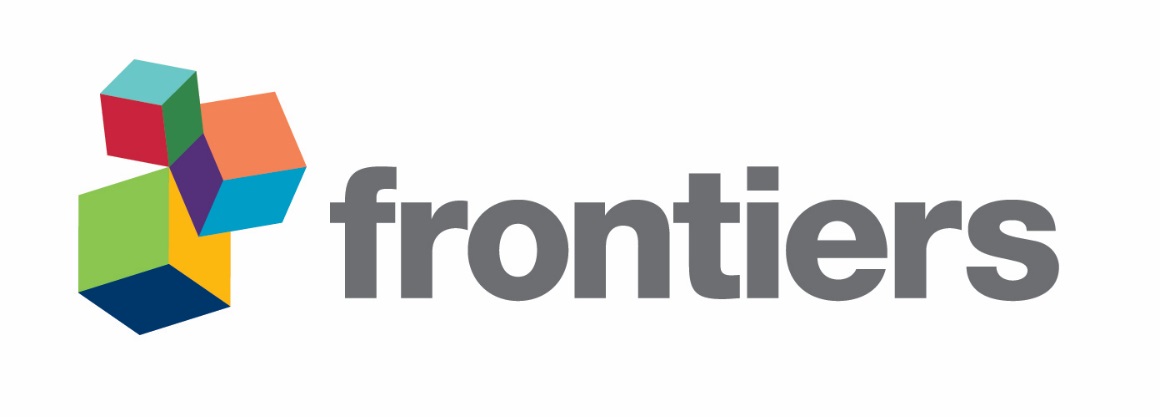
**
