# Supplementary figures and images for: The Raphe Pallidus and the Hypothalamic-Pituitary-Thyroid Axis Gate Seasonal Changes in Thermoregulation in the Hibernating Arctic Ground Squirrel (Urocitellus parryii)
Source: Front Physiol. 2018 Dec 12;9:1747. doi: 10.3389/fphys.2018.01747 (PMC6299024; doi:10.3389/fphys.2018.01747)

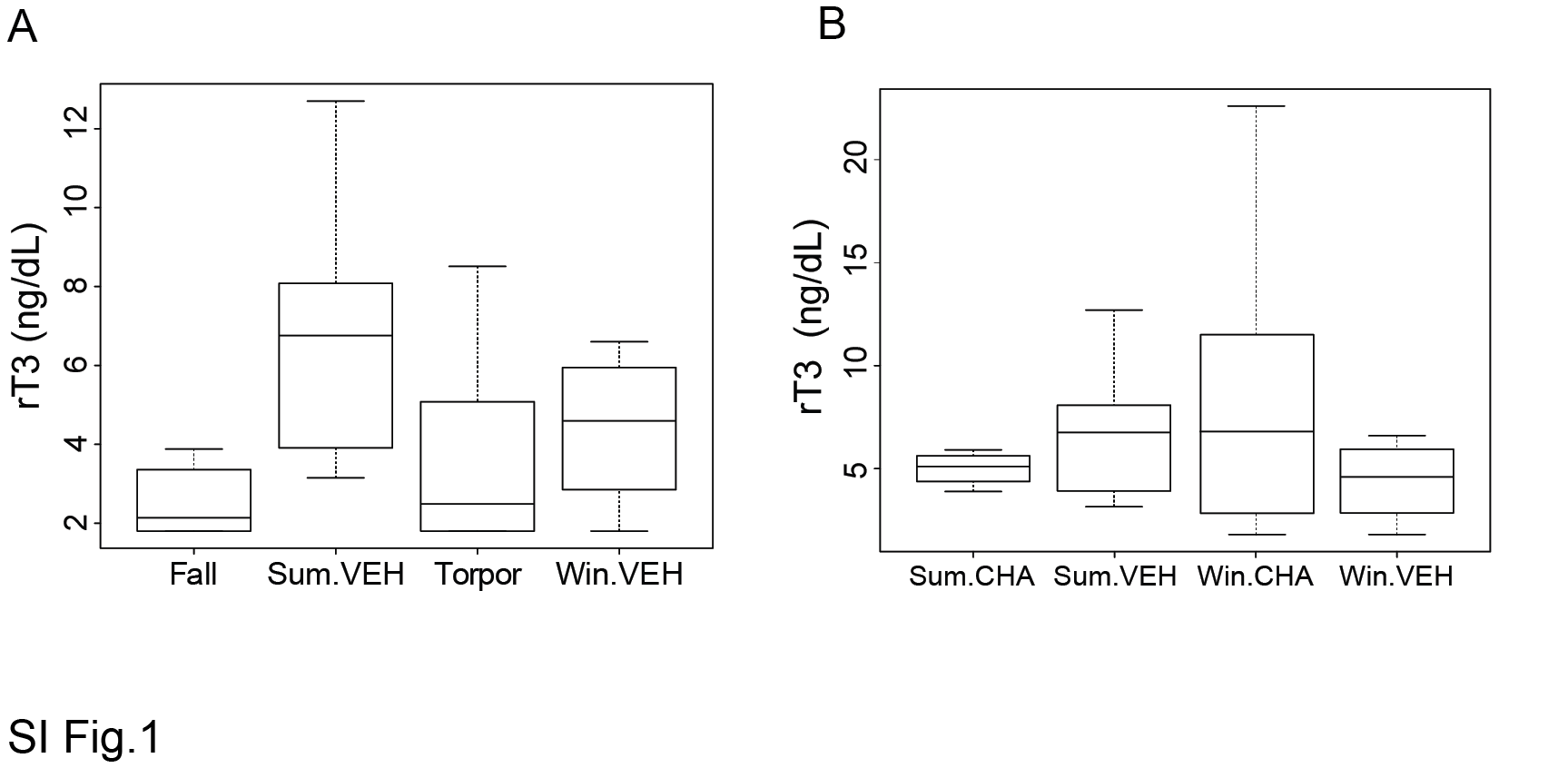

Supplement: Supplementary file 2 [file Image_1.TIF]

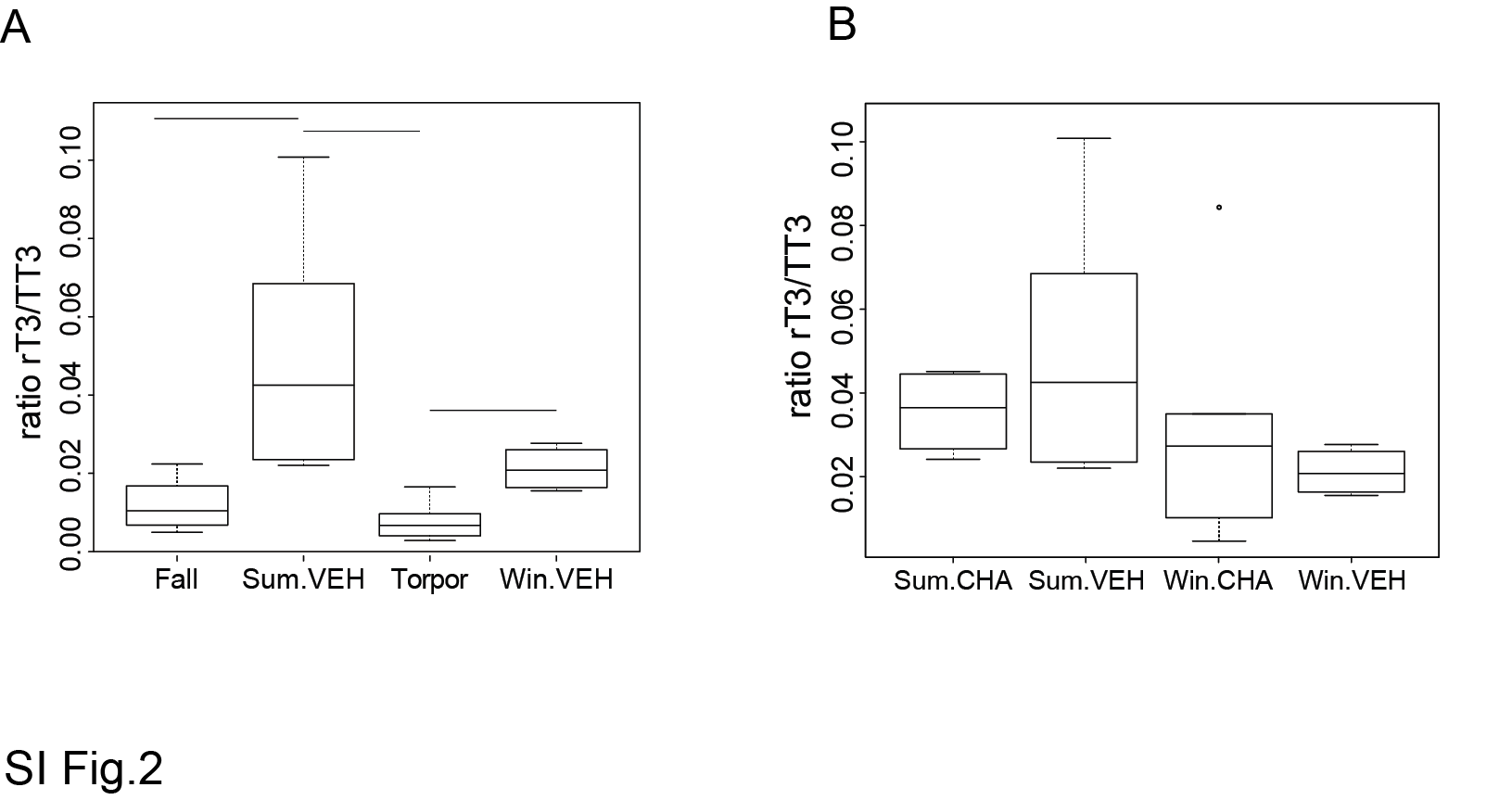

Supplement: Supplementary file 3 [file Image_2.TIF]

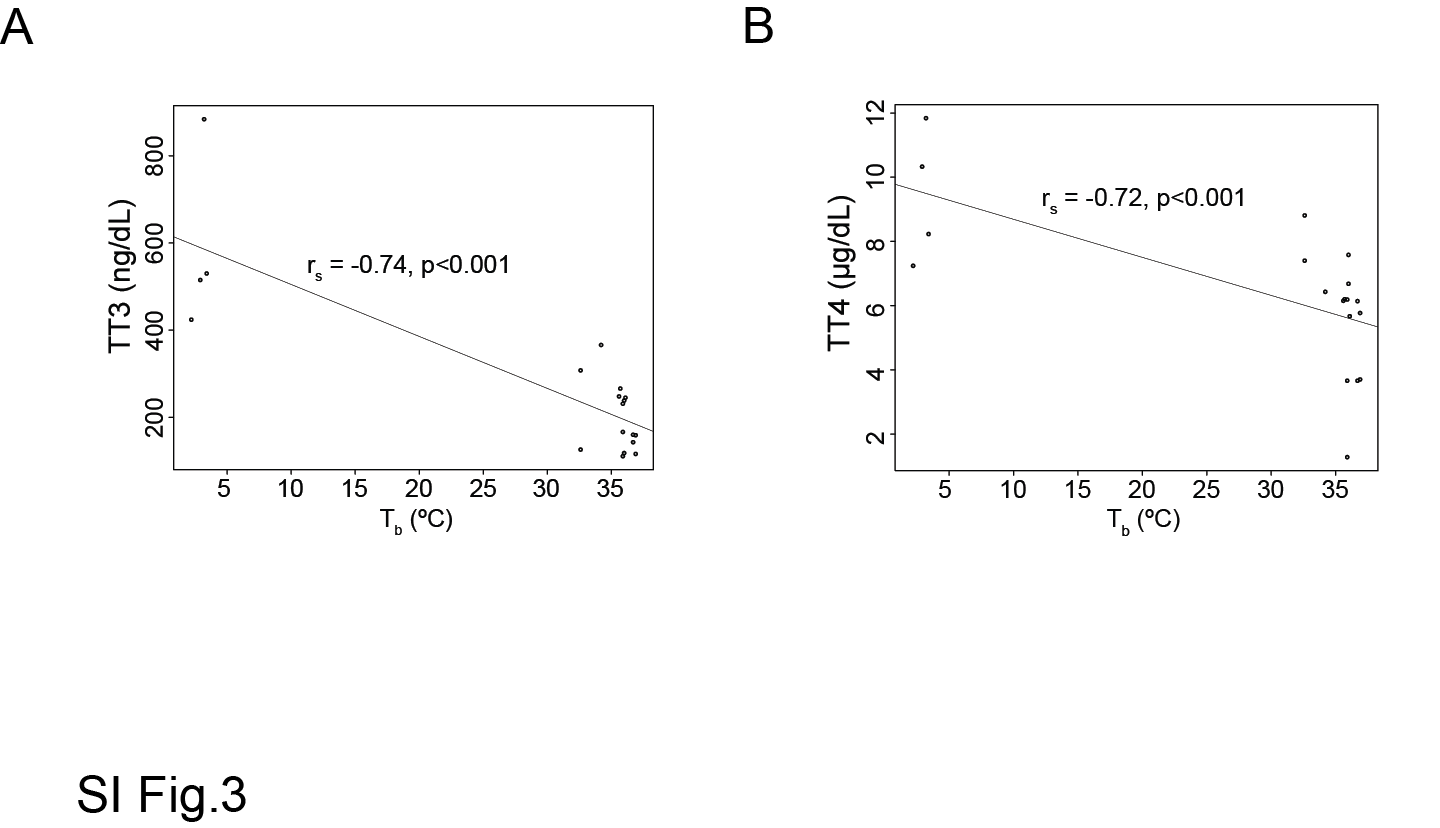

Supplement: Supplementary file 4 [file Image_3.TIF]
